# Aerobic Exercise Preconditioning Does Not Augment Muscle Hypertrophy During Subsequent Resistance Exercise Training in Healthy Older Adults

**DOI:** 10.1007/s40279-025-02229-y

**Published:** 2025-04-23

**Authors:** Milan W. Betz, Alejandra P. Monsegue, Lisanne H. P. Houben, Floris K. Hendriks, Janneau van Kranenburg, Thorben Aussieker, Bouke P. Adriaans, Alfons J. H. M. Houben, Lex B. Verdijk, Luc J. C. van Loon, Tim Snijders

**Affiliations:** 1 Department of Human Biology, NUTRIM Institute of Nutrition and Translational Research in Metabolism, Maastricht University Medical Centre+, P.O. Box 616, 6200 MD Maastricht, The Netherlands; 2 Cardiovascular Research Institute Maastricht (CARIM), Maastricht University, Maastricht, The Netherlands; 3 Department of Internal Medicine, Cardiovascular Research Institute Maastricht (CARIM), Maastricht University, Maastricht, The Netherlands

## Abstract

**Background:**

Resistance exercise training is an effective treatment strategy to counteract the age-related loss of muscle mass and strength in older adults. However, there is a large inter-individual variation in muscle fiber hypertrophy following resistance exercise training. It has been hypothesized that a less than optimal muscle fiber capillarization and perfusion capacity may compromise muscle hypertrophy during resistance exercise training in older adults.

**Objective:**

We assessed whether 8 weeks of aerobic exercise preconditioning, to improve muscle fiber capillarization and perfusion capacity, augments the gains in muscle mass and strength during subsequent resistance exercise training in older adults.

**Methods:**

In total, 34 healthy older males and females (71 years standard deviation (SD) ± 5 years) participated in 12 weeks of progressive resistance exercise training, preceded by either 8 weeks of aerobic preconditioning (AER, *n* = 17) through cycle-ergometer endurance training, or a no exercise control condition (CON, *n* = 17). Muscle strength (one repetition maximum (1RM)) and muscle fiber characteristics (histochemistry) were assessed at baseline, following 8 weeks of AER or CON, and after 12 weeks of resistance exercise training. Femoral artery blood flow and vastus lateralis muscle microvascular perfusion kinetics were assessed at baseline and following 8 weeks of AER or CON intervention. Thigh muscle volume (magnetic resonance imaging scan) was assessed before and after the 12 weeks of resistance exercise training.

**Results:**

Aerobic exercise preconditioning increased type I (+ 19 ± 19%, *P* < 0.05) and type II (+ 35 ± 37%, *P* < 0.05) muscle capillary-to-fiber ratio, with no changes in the CON group (type I: + 0 ± 17%; type II: − 3 ± 26%). Muscle microvascular perfusion following a submaximal resistance exercise stimulus was reduced following aerobic exercise preconditioning, whereas no changes were observed in the CON group (interaction effect, *P* = 0.051). Resistance exercise training increased leg press 1RM (+ 16 ± 10% versus + 12 ± 8%, respectively, *P* < 0.001) and thigh muscle volume (+ 0.42 ± 0.69 versus + 0.31 ± 0.62 L, respectively, *P* < 0.001) in both the AER and CON groups, with no differences between the groups. No differences were observed in type I and type II muscle fiber hypertrophy in response to the entire intervention program between groups (interaction effect, *P* > 0.5).

**Conclusions:**

Aerobic exercise preconditioning increases type I and type II muscle fiber capillarization in healthy older adults. Aerobic exercise preconditioning does not further increase muscle hypertrophy during subsequent resistance exercise training in healthy older adults. Both structural and functional microvascular characteristics do not seem to restrict the skeletal muscle adaptive response to resistance-type exercise training in healthy older adults.

**Supplementary Information:**

The online version contains supplementary material available at 10.1007/s40279-025-02229-y.

## Key Points

**Table:** 

Aerobic exercise preconditioning increases muscle fiber capillarization but not microvascular perfusion in healthy older adults.
Resistance exercise training effectively increases skeletal muscle mass and strength in healthy older adults.
Aerobic exercise preconditioning does not further augment muscle hypertrophy following subsequent resistance exercise training in healthy older adults.

## Introduction

Age-related loss of muscle mass and strength, termed sarcopenia, has been recognized as an independent disease since 2016 [1]. The impact of muscle mass and strength loss on general health is broad, as it is associated with a decline in mobility, higher risk of other diseases (e.g., type 2 diabetes mellitus), lower quality of life, and increased risk of mortality [1]. Resistance exercise training is currently the only effective treatment strategy to counteract sarcopenia. However, when compared with the young, older adults exhibit an attenuated skeletal muscle growth response to prolonged resistance exercise training [2–4]. In addition, large inter-individual variation in muscle mass gains is observed following resistance exercise training in older adults [5]. Recent work suggests that the age-related decline in muscle perfusion capacity may play a key role in the blunted muscle hypertrophy response to resistance exercise training in the older population [6, 7].

Adequate perfusion is essential for muscle tissue maintenance and growth, as it is responsible for delivery of oxygen, nutrients, and growth factors as well as removal of waste products [8]. However, impairments in both macro- and microvascular blood flow have been observed in older adults when compared with young adults, both at rest and during post-exercise recovery [9–14]. A decrease in microvascular blood flow can largely be attributed to the reduction in the surface area of the microvascular bed (i.e., capillaries) in senescent muscle [15–19]. Previously, we [7] and others [6] have shown that muscle fiber hypertrophy is virtually nonexistent following prolonged resistance exercise training in older adults with a relatively low baseline muscle fiber capillarization. Hence, increasing muscle fiber capillarization may be a prerequisite to maximize the muscle growth response during resistance exercise training in older adults.

Although resistance exercise training is an effective strategy to promote muscle mass and strength gains in older adults, its effect on muscle fiber angiogenesis is much less pronounced. Whereas some studies do [20–22], others do not [7, 23] show an increase in muscle fiber capillarization following prolonged resistance exercise training in older adults. In contrast, aerobic exercise training has consistently been shown to increase muscle fiber capillarization, with a similar response in older adults compared with young adults [18, 24, 25]. As such, first performing aerobic exercise training to increase muscle fiber capillarization, thereby “preconditioning” skeletal muscle tissue, may augment the muscle fiber hypertrophy response following subsequent prolonged resistance exercise training in older adults. Therefore, we assessed the impact of 8 weeks of aerobic exercise preconditioning on muscle fiber hypertrophy during a subsequent 12 weeks of resistance exercise training in healthy older adults. We hypothesized that aerobic exercise preconditioning would augment muscle fiber hypertrophy during the subsequent resistance exercise training.

## Methods

### Participants

In total, 34 healthy older men and women aged 65–85 years with a body mass index between 18.5 and 30 kg⋅m^−2^ were recruited to participate in 12 weeks of progressive resistance exercise training, preceded by either 8 weeks of aerobic exercise preconditioning (AER) or no exercise control (CON). Participants’ characteristics are presented in Table 1. During the initial screening, medical history was assessed to exclude individuals with known medical conditions such as cancer, cardiovascular disease, arthritic conditions, neuromuscular problems, renal disorders, pulmonary diseases, and/or hypertension. An oral glucose tolerance test was performed to exclude patients with type II diabetes mellitus [26]. Participants were cleared to perform resistance and aerobic exercise by a cardiologist who examined electrocardiograms taken at rest and during (sub)maximal cycling exercise. All participants were living independently and had not participated in any structured exercise training program within the last 5 years. Participants were informed of the nature and possible risks of the experimental procedures before their written informed consent was obtained. The study was approved by the Medical Ethics Committee of the Maastricht University Medical Centre + , Netherlands (METC 18-060) and complied with the guidelines set out in the most recent version of the Declaration of Helsinki. This study was part of a larger research project registered at the International Clinical Trials Registry Platform (https://trialsearch.who.int) as NTR7681 and was independently monitored by the Clinical Trial Center Maastricht. Sample size was calculated on the basis of a clinically relevant difference of 24 ± 20% [7] increase in type II muscle fiber size during the 12 week resistance exercise training program between the aerobic exercise preconditioning (AER) and no exercise control (CON) group. The sample size was calculated with a power of 90% and a type I error probability of 0.05. Taking into consideration a drop-out rate of 15%, the final number of participants required was 18 (*n* = 9 men and *n* = 9 women) per group.

**Table 1:** Participants’ characteristics

	CON group	AER group
Sex (male/female)	8/9	8/9
Age (years)	71 ± 5	71 ± 5
Height (m)	1.69 ± 0.11	1.70 ± 0.07
Weight (kg)	74 ± 10	73 ± 10
BMI (kg⋅m^−2^)	26 ± 2	26 ± 2
Systolic blood pressure (mmHg)	126 ± 13	134 ± 12
Diastolic blood pressure (mmHg)	70 ± 9	77 ± 10
Fasting plasma glucose concentration (mmol⋅L^−1^)	5.4 ± 0.5	5.4 ± 0.7
2-h plasma glucose concentration (mmol⋅L^−1^)	6.2 ± 1.9	6.3 ± 2.1
HOMA-IR	1.7 ± 1.8	1.8 ± 1.2

Values are mean ± SD or counts

BMI, body mass index; HOMA-IR, homeostasis model assessment of insulin resistance

### Study Design

Following inclusion, participants were randomized to perform either 8 weeks of aerobic preconditioning (AER) or no exercise control (CON). Subsequently, all participants performed 12 weeks of resistance exercise training. Experimental testing was performed at baseline (pre), following 8 weeks of AER or CON (mid), and following 12 weeks of resistance exercise training (post) (Supplemental Fig. 1). All test procedures were performed within a time window of 2 weeks per time point (i.e., pre, mid, or post).

### Aerobic Capacity

Participants performed a peak oxygen uptake (VO_2peak_) test on a calibrated cycle ergometer (Lode Excalibur Sport, Groningen, the Netherlands) while O_2_ consumption and CO_2_ production were captured and measured continuously (Omnical, Maastricht University, the Netherlands). In addition, heart rate was measured during the test with a chest-strap heart rate monitor (Kalenji, France). Following a 1 min warm-up at 30 watts (W), the load was increased by 1 W every 4 s. Participants were instructed to maintain a cadence between 70 and 90 revolutions per minute (rpm). The test was terminated when the cadence dropped below 60 rpm for more than 10 s or when voluntary fatigue was reached. The VO_2peak_ was determined by taking the highest average of three consecutive volume of oxygen (VO_2_) measurements (i.e., 15 s). Aerobic capacity was assessed at baseline (pre), following 8 weeks of aerobic preconditioning or no exercise control (mid), and following 12 weeks of resistance exercise training (post).

### Aerobic Exercise Preconditioning or No Exercise Control

Participants allocated to the AER group performed supervised aerobic exercise three times per week for 8 weeks at the Maastricht University sports center. Exercise sessions were performed on a stationary bike (Technogym, Italy) every Monday and Friday and on a cross-trainer (Technogym, Italy) every Wednesday while wearing a chest-strap heart rate monitor (Kalenji, France). Every exercise session consisted of a 5 min warm-up followed by 45 min at a heart rate corresponding to 65% peak oxygen consumption (VO_2peak_) and a 5 min cool-down. To correct for a higher heart rate during whole-body exercise on the cross-trainer, the target heart rate was increased by 5% of the maximal heart rate. The exercise workload (i.e., wattage) was increased by 5–10 W when the average heart rate during the 45 min protocol was 1 or more beats per min below the target heart rate. Following each session, participants were asked to indicate their rate of perceived exertion on a 6–20 Borg scale. If they indicated 14 or lower while hitting the target heart rate, the target heart rate was increased by 2.5% of the maximal heart rate for the next training session to ensure adequate exercise intensity. Participants in the CON group performed no exercise training during the initial 8 weeks of the study and kept their habitual physical activities as constant as possible.

### Resistance Exercise Training

Following 8 weeks of aerobic preconditioning or no exercise control, all participants performed 12 weeks of supervised resistance exercise training at the Maastricht University sports centre, three times per week (Monday, Wednesday, and Friday). Training sessions consisted of a 5 min warm-up on a stationary bike, followed by both lower and upper body resistance exercises. Lower body exercises (i.e., leg press and leg extension) were performed every session starting with a warm-up set, followed by three sets of eight repetitions and a fourth set taken until voluntary failure. Two upper body exercises, alternating between either chest press and horizontal row or shoulder press and lateral pulldown, were performed in between the two lower body exercises. Upper body exercises started with a warm-up set, followed by one set of eight repetitions and a second set until voluntary failure. The workload was increased from 70 to 75% one-repetition maximum (1RM) in the first week, followed by 80% 1RM in the beginning of the second week. Starting from the second week, weights were increased by 2.5–5 kg when more than ten repetitions were performed in the final set. Participants were provided with at least 90 s of rest between sets and exercises. Each session ended with a 5 min cool-down on a stationary bike. Weekly resistance exercise training volume was calculated as the product of the number of repetitions and weight lifted for all exercises.

### Habitual Dietary Intake and Physical Activity

To assess potential changes in daily food intake throughout the study, all participants completed a 3-day habitual dietary intake record at baseline, during week 7 of the aerobic preconditioning or no exercise control, and during week 11 of the resistance exercise training. Habitual dietary intake records were analyzed for average daily energy intake (megajoule [MJ]), as well as the macronutrient composition of the diet related to protein, carbohydrate, and fat intake, using web-based software (Eetmeter; Voedingscentrum, Den Haag, the Netherlands). Habitual physical activity level was assessed by a hip worn accelerometer (Actical, Philips, the Netherlands) on the same days and time points as the dietary intake records. Data from the accelerometer were analyzed using the Actical software (version 3.10).

### Maximal Muscle Strength

Maximal muscle strength was assessed by 1RM strength tests for the leg press and leg extension exercise (Technogym, Italy). During their first visit, participants were familiarized with all exercises, and 1RM was estimated using the multiple repetitions testing procedure [27]. This estimation was used during the subsequent 1RM tests, as described previously [28]. 1RM was assessed at baseline (pre), following 8 weeks of aerobic preconditioning or no exercise control (mid), and following 12 weeks of resistance exercise training (post).

### Thigh Skeletal Muscle Volume

A magnetic resonance imaging (MRI) scan was performed to assess thigh muscle volume at Scannexus (Brightlands Maastricht Health Campus, Maastricht, the Netherlands) at the mid and post time points. The participant was scanned in a supine position (entering the magnet head first), with knees and hips fully extended, in a 3 T MAGNETOM Prisma Fit scanner using a whole-body coil and an 18-element body array coil (Siemens Healthcare, Erlangen, Germany). A 6 min dual-echo Dixon Vibe protocol was applied, providing a water and fat separated volumetric data set covering head to ankles. Common scanning parameters for all included slabs were: flip angle (alpha) = 10°, repetition time (TR) = 3.89 ms, echo time (TE) = 1.22/2.45 ms, bandwidth = 930 Hz/Px, and 256 × 192 matrix. There was no interslice gap (0 mm). The slabs covering the thighs consisted of 88 slices with a voxel size of 2.0 mm^3^ × 2.0 mm^3^ × 3.0 mm^3^. Image analyses were performed externally by AMRA Medical AB using the AMRA researcher software (AMRA Medical AB, Linköping, Sweden). In brief, image analyses consisted of (1) image calibration, (2) fusion of image stacks, (3) image segmentation, and (4) quantification of muscle volumes and included manual quality control by an analysis engineer [29].

### Body Composition and Anthropometrics

Whole body and regional lean and fat mass were assessed using dual energy X-ray absorptiometry (DXA; Discovery A; Hologic, USA) following a 10–12 h overnight fast. Body mass and height were assessed using a digital scale to the closest 100 g and a fixed stadiometer to the nearest 0.5 cm, respectively. Body composition and anthropometrics were assessed at baseline (pre), following 8 weeks of aerobic preconditioning or no exercise control (mid), and following 12 weeks of resistance exercise training (post).

### Physical Function

Physical functioning was assessed using the short physical performance battery (SPPB) [30], timed up-and-go test (TUG), and handgrip strength test. The SPPB consists of three components: balance, gait speed, and chair-rise ability [30]. The TUG includes a chair rise, a 3 m comfortable walk, turn, walk back, and sit down [31]. Maximal handgrip strength was determined using a hydraulic hand dynamometer (Jamar, Sammons Preston, Bolingbrook, IL, USA). The highest of three consecutive attempts of the dominant and nondominant hand was recorded to the nearest 1 kg with participants sitting in an upright position with the arm at a 90° angle. Physical functioning tests were assessed at baseline (pre), following 8 weeks of aerobic preconditioning or no exercise control (mid), and following 12 weeks of resistance exercise training (post).

### Leg and Muscle Tissue Perfusion Kinetics

Before and after the 8 weeks of no exercise control or aerobic preconditioning, the mean blood velocity and arterial lumen diameter in the common femoral artery were assessed using a high-end Doppler ultrasound machine (Affinity 70G, Philips, the Netherlands) with a linear array probe (eL18-4, Philips, the Netherlands). In addition, in a subgroup (*n* = 7 AER and *n* = 8 CON group), vastus lateralis muscle tissue microvascular perfusion kinetics were assessed using contrast-enhanced ultrasound (CEUS). All measurements were performed in a supine position. The first measurements were performed following 30 min of bed rest, and this was designated as resting condition. First, Doppler ultrasound was used to assess the arterial lumen diameter by video calipers, and the measurement was defined as the maximum distance between the media–adventitia interface of the near wall and the lumen–intima interface of the far wall of the vessel. Measurements were made 2–3 cm proximal to the bifurcation of the femoral artery to minimize the effect of turbulence; the insonation angle was < 60°. To assess mean femoral artery blood flow velocity, 30 s video clips were recorded, and mean blood velocity was assessed for each clip using automatic tracing software. Next, femoral artery blood flow (L·min^−1^) was calculated using the system’s software (formula: blood flow = ((π) × (femoral artery radius)^2^ × (mean blood flow velocity) × (60))). At every time point that femoral artery blood flow was assessed, systolic and diastolic blood pressure were determined to calculate mean arterial pressure using the formula: mean arterial pressure = (diastolic blood pressure + (1/3 × (systolic blood pressure – diastolic blood pressure))) and to calculate femoral artery vascular conductance (ratio of blood flow/mean arterial pressure). Next, vastus lateralis muscle microvascular perfusion kinetics were assessed in the same leg. An ultrasound probe was fixed in a custom-made holder to visualize a cross-sectional image of the vastus lateralis muscle at 1/3 distance between the superior patellar border and the anterior superior iliac spine. This position was marked on the leg using a pen, and a screenshot in ultrasound B-mode was taken to ensure repeated measurements at the exact same position. A 5 s clip was acquired in color Doppler mode to be used during subsequent analysis. Next, an infusion of gas-filled microbubbles (SonoVue, Bracco, concentration: 8 μL⋅mL^−1^) was initiated via a catheter placed in an antecubital vein. For each CEUS measurement, a 10 mL suspension of microbubbles was infused for 6 min at 85 mL⋅h^−1^. Following 3 min of infusion to achieve a steady state of circulating microbubbles, six 30-s recordings were acquired using contrast mode (8 Hz with a mechanical index (MI) of 0.07). At the start of each recording, a high MI flash (0.53 MI) was given to destroy all visible microbubbles, and the subsequent replenishment of microbubbles was recorded. Next, subjects performed a single bout of resistance exercise consisting of four sets of eight repetitions with 80% of 1RM (the value from the test from the week before, i.e., either pre or mid) on the leg press and leg extension machines. In addition, 2 min of rest between sets and 3 min of rest between exercises were allowed. Immediately after exercise, subjects were put into a supine position, and mean femoral artery blood velocity and diameter measurements were repeated at *t* = 5, *t* = 15, *t* = 25, and *t* = 35 min of post-exercise recovery. In addition, vastus lateralis muscle microvascular perfusion was assessed at *t* = 10 and *t* = 40 min of post-exercise recovery. All CEUS recordings were analyzed using ImageJ (version 2.1.0/1.53c), as described previously [32].

### Muscle Biopsy Sampling

Muscle biopsy samples were obtained from the middle region of the vastus lateralis, approximately 15 cm above the patella and 3 cm below entry through the fascia, using the percutaneous needle biopsy technique custom-adapted for manual suction [33]. Muscle samples were dissected carefully and freed from any visible non-muscle material. The biopsy sample was embedded in Tissue-Tek (Sakura Finetek, Zoeterwoude, the Netherlands) and frozen in liquid nitrogen-cooled isopentane. All samples were stored at − 80 °C until further analysis. Participants were instructed to refrain from any strenuous physical activity in the 3 days before the muscle biopsy was taken. In addition, all (maximal) exercise testing was performed at least 5 days before the muscle biopsies were taken. Muscle biopsy samples were obtained at baseline (pre), following 8 weeks of aerobic preconditioning or no exercise control (mid), and following 12 weeks of resistance exercise training (post).

#### Immunohistochemistry

**Fig. 1:**
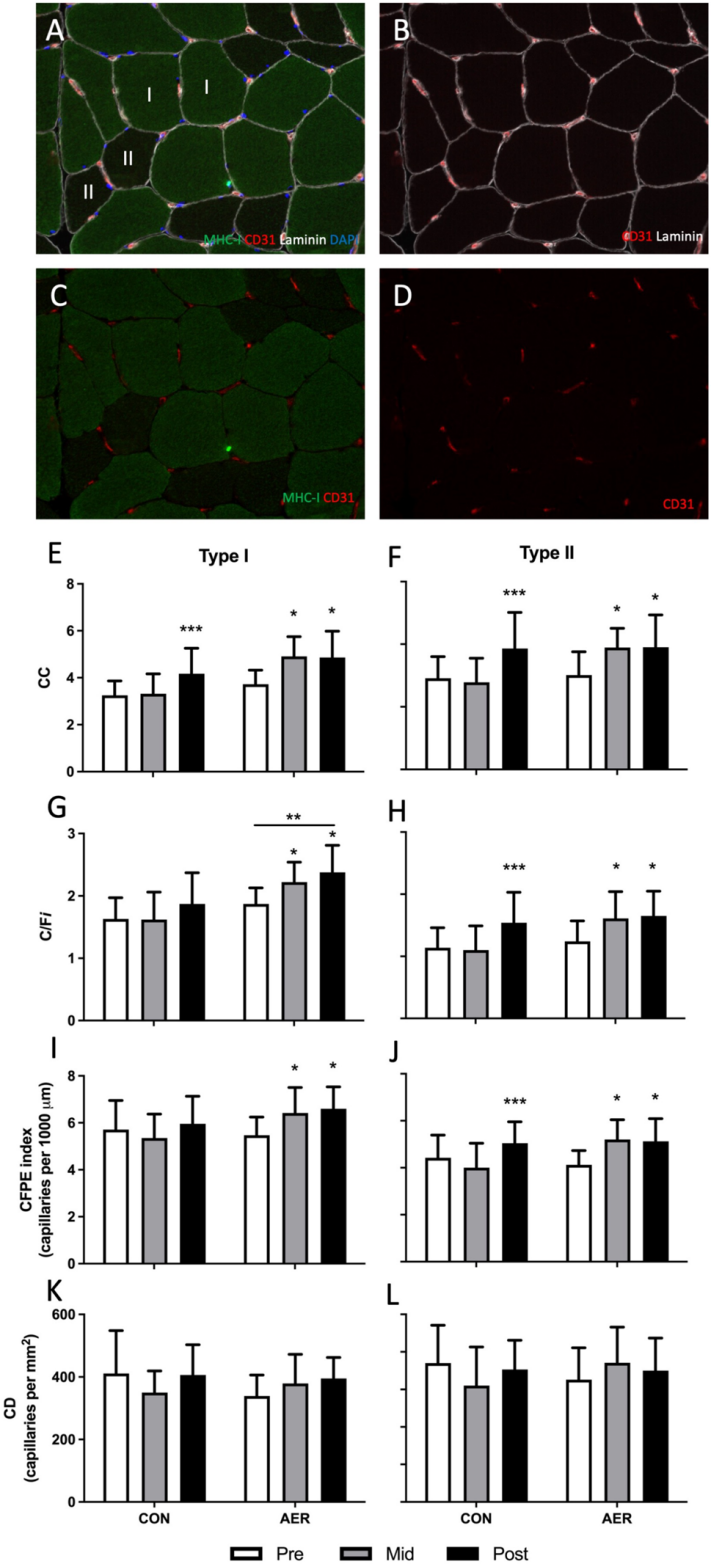
Representative images of the analyses for type I and type II muscle fiber characteristics in older adults; **A** laminin (white; cell borders), MHC1 (green; type I muscle fibers), CD31 (red; capillaries), DAPI (blue; nuclei); **B** laminin (white), CD31 (red); **C** MHC1 (green), CD31 (red); **D** CD31 (red) only. Green fibers indicate type I muscle fibers. Type I and type II capillary contacts (CC, **E–F**), capillary-to-fiber ratio (C/F*i*, **G–H**), capillary-to-fiber perimeter exchange (CFPE) index (**I–J**), and capillary density (CD, **K–L**) at baseline (pre), following 8 weeks of aerobic exercise preconditioning (AER, mid) or no exercise training (CON, mid), and following 12 weeks of subsequent resistance exercise training (post) in healthy older adults. *Significantly different compared with pre, *P* < 0.05; **significantly different compared with CON group, *P* < 0.05; ***significantly different compared with pre and mid, *P* < 0.05; data are expressed as mean ± SD

Frozen muscle biopsies were cut into 5-μm-thick cryosections using a cryostat at − 20 °C and thaw-mounted onto uncoated glass slides. Care was taken to properly align the samples for cross-sectional orientation of the muscle fibers. Primary antibodies CD31 (dilution 1/50; M0823; Dako, Glostrup, Denmark), myosin heavy chain (MHC) -I (BA-F8, dilution 1/10; DSHB), laminin (polyclonal rabbit anti-laminin, dilution 1/50; Sigma) with appropriate secondary antibodies Avidine Texas Red (A2006, dilution 1/400; Vector Laboratories), goat anti-mouse (GAM) IgG2b AlexaFluor488, goat anti-rabbit (GAR) IgG AlexaFluor647 (Molecular Probes), and DAPI were used to stain for capillaries, type I muscle fibers, laminin, and nuclei, respectively, as described previously (see Fig. 1) [32]. Slides were viewed and automatically captured using 20X objective on a modified Olympus BX51 fluorescence microscope with a customized spinning disk unit (DSU, Olympus), computer-controlled excitation and emission filter wheels (Olympus), 3-axis high-accuracy computer-controlled stepping motor specimen stage (Grid Encoded Stage, Ludl Electronic Products, Hawthorne, NY, USA), ultra-high sensitivity monochrome electron multiplier CCD camera (C9100-02, Hamamatsu Photonics, Hamamatsu City, Japan), and controlling software (StereoInvestigator; MBF BioScience, Williston, VT, USA). Quantitative analyses were performed using ImageJ version 2.1.0/1.53c. On average, 122 ± 52 (pre), 110 ± 46 (mid), and 107 ± 57 (post) muscle fibers were analyzed per fiber type per participant to determine muscle fiber type distribution, size, and myonuclear content. The quantification of muscle fiber capillaries was performed on 30 type I and 30 type II muscle fibers on the basis of the work of Hepple et al. [34], to determine (i) capillary contacts (CC), (ii) the capillary-to-fiber ratio (C/F*i*), (iii) capillary-to-fiber perimeter exchange (CFPE) index in capillaries per 1000 µm, and (iv) capillary density (CD) in capillaries per mm^2^. All immunofluorescence analyses were completed in a blinded fashion.

### Statistical Analyses

All data are expressed as mean ± SD. Changes over time were analyzed using a two-factor repeated measures analysis of variance (ANOVA) with time (pre versus mid versus post) as within-subject factor and treatment group (AER versus CON) as between-subject factor for the analysis of muscle fiber characteristics, 1RM, aerobic capacity, body composition, physical functioning, dietary intake, physical function, and activity level. In cases where there was a significant interaction, a one-way ANOVA was performed to locate differences over time, while Student’s unpaired *t*-tests were used to locate differences between groups. Changes in thigh muscle volume were analyzed using a two-factor repeated measures ANOVA with time (mid versus post) as within-subject factor and treatment group (AER versus CON) as between-subject factor. Femoral artery diameter, blood flow/velocity, vascular conductance, blood pressure, and heart rate at rest and following a single bout of resistance exercise were analyzed using a three-way repeated measures ANOVA with intervention time (pre versus mid) and acute exercise time (rest versus *t* = 5 versus *t* = 15 versus *t* = 25 versus *t* = 35 min) as within-subject factors and treatment group (AER versus CON) as between-subject factor. Vastus lateralis muscle microvascular blood volume, velocity, and flow were analyzed using a three-way repeated measures ANOVA with intervention time (pre versus mid) and acute exercise time (rest versus *t* = 10 versus *t* = 40 min) as within-subject factors and treatment group (AER versus CON) as between-subject factor. Changes in weekly resistance exercise training volume were analyzed using a two-factor repeated measures ANOVA with time (week 1–12) as within-subject factor and treatment group (AER versus CON) as between-subject factor. The Bonferroni correction was used to correct for multiple comparisons. Statistical significance was set at *P* < 0.05, and all calculations were performed using SPSS (version 24.0; IBM Corporation).

## Results

### Subject Characteristics

The study population included nine females and eight males in each of the CON and AER groups. Age, height, weight, and body mass index (BMI) were not significantly different between the groups at baseline (Table 1). Resting systolic and diastolic blood pressure ranged from 101 to 158 mmHg and from 54 to 89 mmHg, respectively. On the basis of fasting plasma glucose values, 79% of all participants had a normal and 21% had impaired fasting glucose (< 5.6 versus > 5.6 mmol·L^−1^, respectively). On the basis of the plasma glucose concentrations at the 2-h time point during the oral glucose tolerance test (OGTT), 76% of participants showed normal (< 7.8 mmol·L^−1^) glucose tolerance and 24% had impaired glucose tolerance (> 7.8 mmol·L^−1^; Table 1). None of the participants were categorized as having diabetes on the basis of 2-h glucose concentrations (> 11.1 mmol·L^−1^).

### Muscle Fiber Capillarization

For both type I and type II muscle fiber CC, a significant *time* × *group* interaction was observed (both *P* < 0.05, Fig. 1E, F). Post hoc within-group analyses showed a significant increase in type I and type II muscle fiber CC following 8 weeks of exercise preconditioning (*P* < 0.05 for both type I and type II muscle fibers in the AER group), with no further changes during the subsequent 12 weeks of resistance exercise training. In contrast, the CON group showed no changes in type I and type II muscle fiber CC in the initial 8 weeks (no exercise control condition), followed by a significant increase during the subsequent 12 weeks of resistance exercise training (*P* < 0.05 for both type I and type II muscle fibers, Fig. 1E, F). For both type I and type II muscle fibers C/F*i* (Fig. 1G, H), as well as CFPE-index (Fig. 1I, J), *time* × *group* interactions were observed (all *P* < 0.05). Whereas no significant changes were observed for type I muscle fiber C/F*i* and CFPE-index over all three time points in the CON group, type I muscle fiber C/F*i* and CFPE-index increased significantly during the first 8 weeks of exercise preconditioning in the AER group (Fig. 1G–J, *P* < 0.05), with no further changes during the subsequent 12 weeks of resistance exercise training. In the CON group, type II muscle fiber C/F*i* and CFPE-index remained unchanged during the first 8 weeks of no exercise control intervention but increased significantly during the subsequent 12 weeks of resistance exercise training (Fig. 1G–J, *P* < 0.05). In the AER group, type II muscle fiber C/F*i* and CFPE-index increased significantly during the initial 8 weeks of exercise preconditioning (*P* < 0.05), with no further changes during the subsequent 12 weeks of resistance exercise training (Fig. 1G–J). For type I and type II muscle fiber CD, a significant *time* × *group* interaction was observed (both *P* < 0.05). Whereas no changes were observed during the first 8 weeks of the intervention program, type I muscle fiber CD tended to increase during the 12 weeks of resistance exercise training in both the CON (*P* = 0.050) and AER (*P* = 0.081) groups (Fig. 1K, L). For type II muscle fibers, a tendency (*P* = 0.069) was observed for a decline in CD in the CON group during the first 8 weeks of no exercise control condition, with no further changes during the subsequent 12 weeks of resistance exercise training. In the AER group, type II muscle fiber CD remained unchanged over the entire exercise training program intervention (Fig. 1K, L).

### Femoral Artery Blood Flow

Femoral artery diameter and blood flow parameters assessed using Doppler ultrasound at rest, and at *t* = 5, *t* = 15, *t* = 25, and *t* = 35 min following a single bout of resistance exercise are presented in Table 2. There were no detectable changes in femoral artery diameter in response to a single bout of exercise compared with the resting condition in both groups at baseline. In addition, the femoral artery diameter at rest and following a single bout of resistance exercise remained unchanged following the first 8 weeks of the intervention program in both the CON and AER groups (Table 2). Femoral artery blood velocity and flow increased significantly in response to a single bout of resistance exercise, peaking at *t* = 5 min post exercise, and returning to resting value at *t* = 35 min in both groups at baseline. The femoral artery blood velocity and blood flow response to the single bout of resistance exercise remained unchanged following the first 8 weeks of the intervention program in both the CON and AER groups (Table 2). Similar results were observed when femoral artery blood flow was corrected for mean arterial blood pressure, expressed as vascular conductance. In response to the single bout of exercise, vascular conductance increased significantly, peaking at *t* = 5 min post exercise, and returning to resting value at *t* = 25 and *t* = 35 min in both groups at baseline. In addition, this response remained unchanged following the first 8 weeks of the intervention program in both the CON and AER groups (Table 2).

**Table 2:** Femoral artery blood flow and blood pressure at rest and at 5 (*t* = 5), 15 (*t* = 15), 25 (*t* = 25), and 35 (*t* = 35) min following a single bout of resistance exercise at baseline (pre) and following 8 weeks (mid) of aerobic exercise preconditioning (AER) or no exercise training (CON) in healthy older adults

	Group	Pre (*n* = 15)					Mid (*n* = 15)				
		Rest	*t* = 5	*t* = 15	*t* = 25	*t* = 35	Rest	*t* = 5	*t* = 15	*t* = 25	*t* = 35
**Femoral artery**											
Diameter (mm)	*CON*	6.4 ± 0.6^a^	6.4 ± 0.6^a^	6.4 ± 0.7^a^	6.4 ± 0.6^a^	6.4 ± 0.6^a^	6.4 ± 0.8^a^	6.3 ± 0.7^a^	6.3 ± 0.7^a^	6.3 ± 0.8^a^	6.2 ± 0.8^a^
	*AER*	6.7 ± 0.8^a^	6.7 ± 0.9^a^	6.7 ± 0.9^a^	6.7 ± 0.6^a^	6.7 ± 0.9^a^	6.7 ± 0.9^a^	6.7 ± 6.9^a^	6.7 ± 0.9^a^	6.7 ± 0.9^a^	6.7 ± 0.9^a^
Velocity (mL)	*CON*	19 ± 7^a^	27 ± 6^b^	22 ± 5^c^	20 ± 5^d^	19 ± 4^ae^	18 ± 5^a^	27 ± 8^b^	23 ± 7^c^	21 ± 7^d^	21 ± 8^ae^
	*AER*	18 ± 7^a^	30 ± 10^b^	24 ± 8^c^	22 ± 7^d^	20 ± 7^ae^	20 ± 5^a^	30 ± 8^b^	26 ± 7^c^	22 ± 4^d^	21 ± 5^ae^
Flow (mL·min^−1^)	*CON*	339 ± 133^a^	512 ± 115^b^	436 ± 173^c^	401 ± 136^d^	379 ± 132^ae^	359 ± 136^a^	506 ± 155^b^	442 ± 142^c^	397 ± 148^d^	391 ± 206^ae^
	*AER*	372 ± 189^a^	643 ± 305^b^	524 ± 267^c^	474 ± 239^d^	432 ± 191^ae^	425 ± 136^a^	655 ± 313^b^	553 ± 246^c^	482 ± 164^d^	454 ± 158^ae^
VC (mL·min^−1^·mmHg^−1^)	*CON*	3.3 ± 1.3^a^	4.7 ± 1.2^b^	4.2 ± 1.2^bc^	3.8 ± 1.3^ad^	3.6 ± 1.3^ade^	3.3 ± 1.3^a^	5.4 ± 3.9^b^	4.1 ± 1.3^bc^	3.7 ± 1.4^ad^	3.7 ± 2.0^ade^
	*AER*	3.3 ± 1.8^a^	5.6 ± 2.8^b^	5.6 ± 2.8^bc^	4.3 ± 2.4^ad^	3.9 ± 1.8^ade^	3.7 ± 1.3^a^	5.7 ± 2.9^b^	5.1 ± 2.3^bc^	4.3 ± 1.6^ad^	4.1 ± 1.5^ade^
**Blood pressure**											
Systolic (mmHg)	*CON*	125 ± 15^a^	129 ± 14^a^	122 ± 15^a^	124 ± 14^a^	129 ± 13^a^	130 ± 17^a^	126 ± 33^a^	126 ± 14^a^	128 ± 14^a^	128 ± 16^a^
	*AER*	137 ± 17^a^	137 ± 9^a^	132 ± 9^a^	131 ± 10^a^	133 ± 10^a^	138 ± 15^a^	136 ± 9^a^	130 ± 10^a^	132 ± 11^a^	131 ± 10^a^
Diastolic (mmHg)	*CON*	69 ± 7^a^	72 ± 7^a^	70 ± 8^a^	71 ± 9^a^	70 ± 7^a^	73 ± 10^a^	75 ± 10^a^	74 ± 9^a^	74 ± 11^a^	73 ± 10^a^
	*AER*	75 ± 8^a^	76 ± 7^a^	74 ± 8^a^	74 ± 7^a^	74 ± 8^a^	73 ± 8^a^	76 ± 6^a^	72 ± 7^a^	75 ± 9^a^	72 ± 8^a^
Heart rate (bpm)	*CON*	61 ± 5^a^	74 ± 9^b^	71 ± 7^c^	68 ± 8^d^	66 ± 8^e^	62 ± 7^a^	73 ± 11^b^	72 ± 8^c^	69 ± 9^d^	67 ± 8^e^
	*AER*	59 ± 5^a^	76 ± 9^b^	71 ± 8^c^	68 ± 8^d^	66 ± 9^e^	59 ± 7^a^	74 ± 9^b^	72 ± 7^c^	67 ± 8^d^	65 ± 8^e^

Data represent mean ± SD

Different letters denote significant within-group difference between time points, *P* < 0.05. No between-group differences were observed. Femoral artery blood flow measurements of *n* = 4 participants (CON *n* = 2, AER *n* = 2) were lost owing to equipment failure

VC, vascular conductance

### Microvascular Perfusion Kinetics

**Fig. 2:**
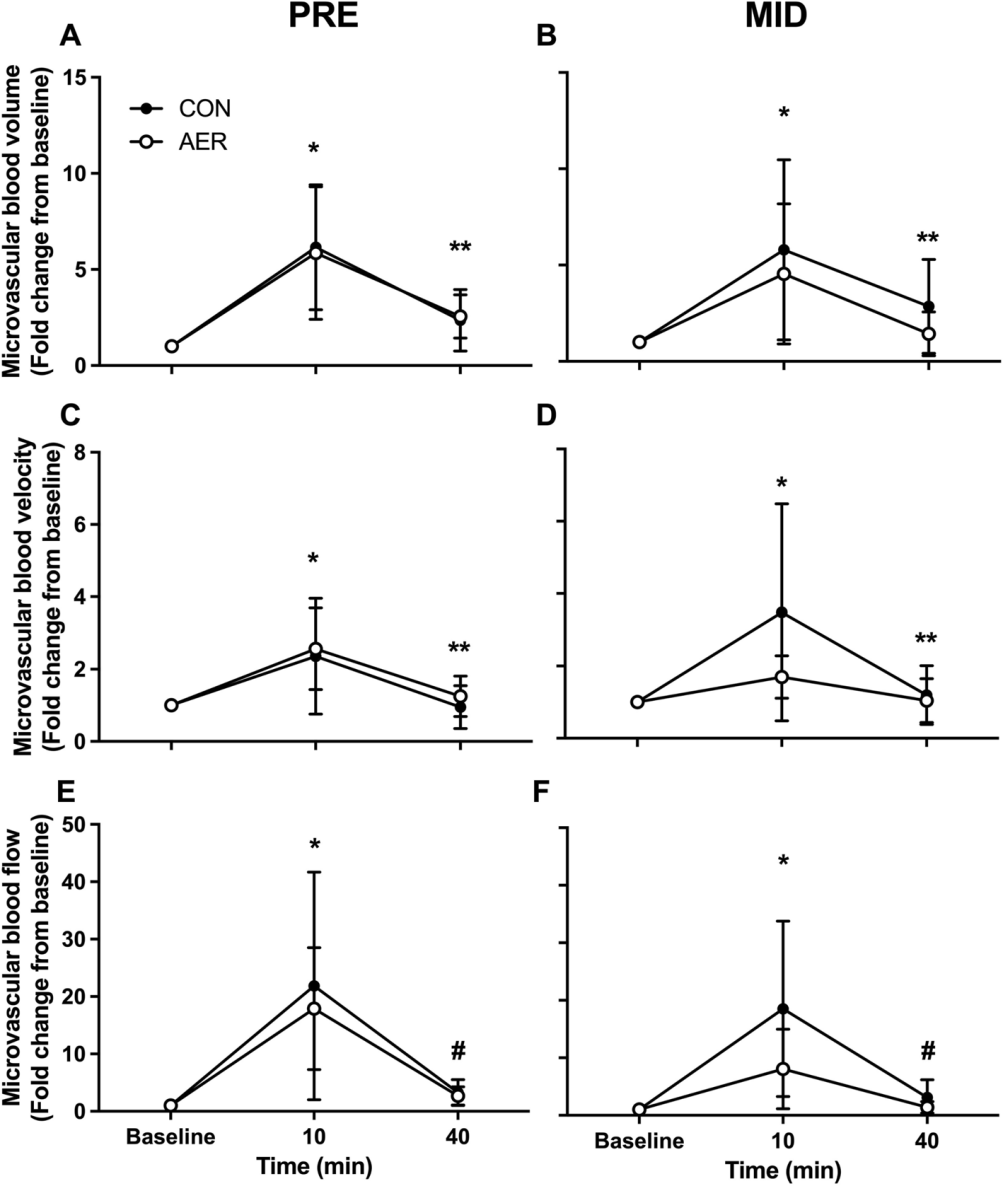
Microvascular blood volume (**A**–**B**), velocity (**C**–**D**), and flow (**E**–**F**), assessed by contrast-enhanced ultrasound, at baseline (rest), and *t* = 10 and *t* = 40 min after a single bout of resistance exercise, before (pre) and after (mid) 8 weeks of aerobic exercise preconditioning (AER, *n* = 7) or no exercise training (CON, *n* = 8), in healthy older adults. Data are expressed as fold change from baseline with mean ± SD. *Significantly different compared with baseline and *t* = 40 min, *P* < 0.05; **significantly different compared with baseline and *t* = 10 min, *P* < 0.05; ^#^significantly different compared with *t* = 10 min, *P* < 0.05

Microvascular perfusion kinetics of the vastus lateralis muscle assessed using CEUS at rest and at *t* = 10 and *t* = 40 min following a single bout of resistance exercise at an intensity of 80% of 1RM are shown in Fig. 2. Microvascular blood volume increased significantly at *t* = 10 min after exercise cessation, and remained elevated until *t* = 40 min, in both the CON (6.2 ± 3.3-fold and 2.8 ± 1.6-fold, respectively) and AER (5.8 ± 3.6-fold and 2.2 ± 1.4-fold, respectively) groups at baseline. Following the initial 8 weeks of the intervention program, the microvascular blood volume response following the single bout of exercise tended (*P* = 0.051 interaction effect) to be lower in the AER group (*t* = 10 min: 4.5 ± 3.6-fold and *t* = 40 min: 1.4 ± 1.1-fold increase from rest), whereas the CON group showed no difference in its response (*t* = 10 min: 5.8 ± 4.7-fold and *t* = 40 min: 2.9 ± 2.4-fold increase from rest; Fig. 2B). Microvascular blood velocity was significantly increased at *t* = 10 min after exercise cessation and returned back to baseline at *t* = 40 min, with no differences between the CON (2.4 ± 1.6-fold and 1.2 ± 0.6-fold, respectively) and AER (2.6 ± 1.1-fold and 1.1 ± 0.6-fold, respectively) groups at baseline (Fig. 2C). Following the first 8 weeks of the intervention program, no significant changes were observed in the post-exercise microvascular blood velocity response for either group (Fig. 2D). Microvascular blood flow showed a significant increase at *t* = 10 min after exercise cessation, and remained elevated until *t* = 40 min in both the CON (21.8 ± 19.3-fold and 3.3 ± 2.3-fold, respectively) and AER (17.9 ± 10.6-fold and 2.7 ± 1.6-fold, respectively) groups at baseline (Fig. 2E). Following the initial 8 weeks of the intervention program, the microvascular blood flow response following a single bout of exercise tended (*P* = 0.061 interaction effect) to be lower in the AER group (*t* = 10 min: 8.1 ± 6.9-fold and *t* = 40 min: 1.4 ± 1.0-fold increase from rest), whereas the CON group showed no difference in its response (*t* = 10 min: 18.5 ± 15.3-fold and *t* = 40 min: 3.1 ± 3.1-fold increase from rest; Fig. 2E, F).

### Muscle Fiber Size, Leg Lean Mass, and Thigh Volume

**Fig. 3:**
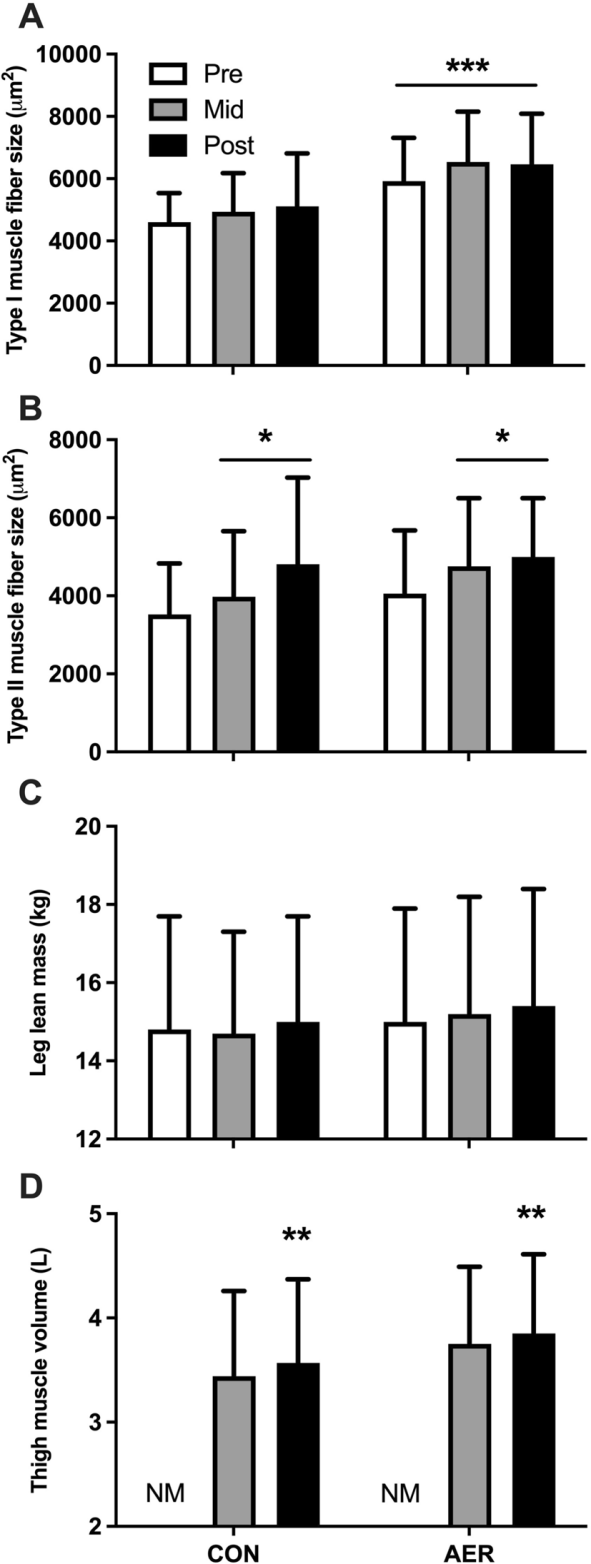
Skeletal muscle hypertrophy measurements based on histology of muscle biopsy samples (**A**–**B**), DXA scanning (**C**), and MRI scanning (**D**) performed at baseline (pre) and following 8 weeks of aerobic exercise preconditioning (AER, mid) or no exercise training (CON, mid) and following 12 weeks of subsequent resistance exercise training (post) in healthy older adults. *Significantly different compared with pre, *P* < 0.05; **significantly different compared with mid, *P* < 0.05; ***significantly different compared with CON group, *P* < 0.05; data are expressed as mean ± SD. NM, no measurement

Type I muscle fiber size remained unchanged over time from pre to mid to post in both the CON and AER groups (Fig. 3C). A main effect of time (*P* < 0.05) was observed for type II muscle fibers, with no significant *time* × *group* interaction effect. Type II muscle fibers were significantly larger at the mid and post time points when compared with baseline (Fig. 3B). A significant main effect of time was observed for leg lean mass (*P* < 0.05), with no *time* × *group* interaction effect. However, post hoc analysis was unable to detect a significant change in leg lean mass during the initial 8 weeks of the intervention program or in response to the subsequent 12 weeks of resistance exercise training (Fig. 3C). Thigh muscle volume, assessed using MRI-scan, was only performed at the mid and post intervention program time points. At the mid time point, thigh muscle volume was not different between the CON and AER groups (3.44 ± 0.82 L versus 3.75 ± 0.74 L, respectively, Fig. 3D). Thigh muscle volume increased significantly in response to 12 weeks of resistance exercise training, with no difference between the CON and AER groups (Fig. 3D).

### Muscle Fiber Type Distribution, Myonuclear Content, and Domain Size

Muscle fiber type distribution remained unchanged over time in both the CON and AER groups (Table 3). Type I muscle fiber myonuclear content remained unchanged over time in both the CON and AER groups. A tendency for a main effect of time (*P* = 0.082) was observed for type II muscle fiber myonuclear content, with no *time* × *group* interaction effect (Table 3). Type I and type II muscle fiber myonuclear domain size were not different between the CON and AER groups and did not change over time in both groups (Table 3).

**Table 3:** Muscle fiber characteristics at baseline (pre), following 8 weeks of aerobic exercise preconditioning (AER, mid) or no exercise training (CON, mid), and following 12 weeks of subsequent resistance exercise training (post) in healthy older adults

	Fiber type	CON group (*n* = 16)			AER group (*n* = 16)		
		Pre	Mid	Post	Pre	Mid	Post
Fiber type distribution (% fiber)	I	43 ± 9	47 ± 15	42 ± 14	49 ± 13	47 ± 13	50 ± 14
	II	57 ± 9	53 ± 15	58 ± 14	51 ± 13	43 ± 13	50 ± 14
Fiber type distribution (% CSA)	I	50 ± 13	53 ± 14	44 ± 15	59 ± 15	55 ± 17	56 ± 16
	II	50 ± 13	47 ± 14	56 ± 15	41 ± 15	45 ± 17	44 ± 16
Myonuclear content (*n* per fiber)	I*	1.92 ± 0.41	2.01 ± 0.40	2.09 ± 0.48	2.42 ± 0.45	2.60 ± 040	2.58 ± 0.57
	II	1.88 ± 0.46	2.02 ± 0.40	2.17 ± 0.56	2.03 ± 0.56	2.29 ± 0.51	2.26 ± 0.55
Myonuclear domain size (µm^2^)	I	2419 ± 282	2451 ± 321	2444 ± 460	2454 ± 337	2524 ± 504	2519 ± 417
	II	1937 ± 384	1939 ± 367	2189 ± 694	1949 ± 274	2050 ± 422	2203 ± 345

Values are mean ± SD

*Significant main effect of group. Muscle biopsy samples of *n* = 2 participants (CON *n* = 1, AER *n* = 1) were of insufficient quality and/or size to perform the histological analysis

CSA, cross-sectional area

### Body Composition

For whole body fat mass, a significant main effect of time (*P* < 0.05) with a tendency for a *time* × *group* interaction (*P* = 0.060) was observed. Whole body fat mass declined during the initial 8 weeks of the intervention program, with no further change during the subsequent 12 weeks of resistance exercise training. We did not detect changes in whole body (main effect of time; *P* = 0.124), appendicular lean mass (main effect of time; *P* = 0.132) in response to the intervention program in both the CON and AER groups (Table 4).

**Table 4:** Body composition at baseline (pre), following 8 weeks of aerobic exercise preconditioning (AER, mid) or no exercise training (CON, mid), and following 12 weeks of subsequent resistance exercise training (post) in healthy older adults

	CON group *n* = 16			AER group *n* = 16		
	Pre	Mid	Post	Pre	Mid	Post
**Body composition**						
Whole body fat mass (kg)	23.4 ± 4.2	23.2 ± 3.9*	23.2 ± 4.3	23.0 ± 3.6	22.2 ± 3.9*	22.1 ± 3.7
Whole body lean mass (kg)	47.5 ± 8.5	47.6 ± 8.1	47.9 ± 8.5	47.3 ± 8.5	47.3 ± 8.3	47.7 ± 8.7
Appendicular lean mass (kg)	20.1 ± 4.1	20.0 ± 3.7	20.2 ± 2.7	20.5 ± 4.4	20.5 ± 4.3	20.7 ± 3.9

Values are mean ± SD

*Significantly different compared with pre, *P* < 0.05. Body composition measurements of *n* = 2 participants (CON *n* = 1, AER *n* = 1) were lost owing to equipment failure

### Muscle Strength, Aerobic Capacity, and Physical Function

Weekly resistance exercise training volume increased over time (*P* < 0.001), with no difference between the CON and AER groups (data not shown). No significant *time* × *group* interactions were observed for any of the 1RM outcomes. For both 1RM muscle strength outcomes, a significant main effect of time was observed (*P* < 0.05). Whereas 1RM muscle strength remained unchanged during the first 8 weeks of the intervention program, 1RM increased significantly during 12 weeks of resistance exercise training for all exercises (all *P* < 0.05, Table 5).

**Table 5:** Muscle strength, aerobic capacity, and physical function at baseline (pre), following 8 weeks of aerobic exercise preconditioning (AER, mid) or no exercise training (CON, mid), and following 12 weeks of subsequent resistance exercise training (post) in healthy older adults

	CON group (*n* = 17)			AER group (*n* = 17)		
	Pre	Mid	Post	Pre	Mid	Post
**Muscle strength**						
1RM leg press (kg)	145 ± 35	143 ± 31	161 ± 36**	148 ± 41	145 ± 40	167 ± 46**
1RM leg extension (kg)	67 ± 19	68 ± 19	79 ± 21**	72 ± 15	75 ± 17	88 ± 22**
**Aerobic capacity**						
Absolute VO_2peak_ (L⋅min^−1^)	2.17 ± 0.56	2.14 ± 0.56	2.23 ± 0.54**	2.15 ± 0.46	2.27 ± 0.41*	2.22 ± 0.46
Relative VO_2peak_ (mL⋅min^−1^⋅kg^−1^)	29.3 ± 5.6	29.5 ± 6.0	30.6 ± 5.7**	29.6 ± 5.6	32.0 ± 5.5*	31.4 ± 5.9
Peak aerobic power (W)	171 ± 52	172 ± 55	179 ± 56	184 ± 45	198 ± 45	193 ± 52
**Physical function**						
SPPB (points)	11.5 ± 0.6	11.2 ± 0.9	11.5 ± 0.6	11.5 ± 0.6	11.4 ± 0.7	11.5 ± 0.5
TUG (s)	7.8 ± 1.3	8.0 ± 1.3	8.1 ± 1.6	7.8 ± 0.8	8.0 ± 0.8	8.1 ± 0.9
Handgrip strength (kg)	33 ± 10	34 ± 10	35 ± 10*	33 ± 9	34 ± 9	35 ± 8*

Values are mean ± SD

*Significantly different compared with pre, *P* < 0.05; ** significantly different compared with mid, *P* < 0.05

1RM, one-repetition maximum; VO_2peak_, peak oxygen consumption; SPPB, short physical performance battery (max score is 12 points); TUG, time up-and-go test

For peak aerobic power output, a tendency was observed for a main effect of time (*P* = 0.054) and *time* × *group* interaction (*P* = 0.078). Both absolute and relative VO_2peak_ (relative to bodyweight) showed a significant *time* × *group* interaction effect (both *P* < 0.05). Whereas no changes were observed in the CON group, absolute and relative VO_2peak_ increased significantly in response to the 8-week aerobic preconditioning in the AER group (both *P* < 0.05). During the subsequent 12 weeks of resistance exercise training, absolute and relative VO_2peak_ increased significantly in the CON group (both *P* < 0.05), whereas no further change was observed in the AER group (Table 5).

SPPB-score remained unchanged in response to the intervention program in both groups. A tendency (*P* = 0.062) for a main effect of time was observed for TUG, with no difference between the two groups. A significant main effect of time (*P* < 0.05) was observed for handgrip strength, with a significant increase observed after 12 weeks of resistance exercise training compared with baseline in both groups (Table 5).

### Habitual Dietary Intake and Physical Activity Level

Energy, fat, and carbohydrate intake remained unchanged over time in both the CON and AER groups. For protein intake, expressed as absolute amount and relative to bodyweight, no changes were observed over time in both groups (Table 6). Number of steps taken per day, habitual sedentary time, and time spent performing light activity remained unchanged over time. A main effect of time was observed for habitual total energy expenditure (Table 6, *P* < 0.05). Whereas habitual total energy expenditure remained unchanged during the initial 8 weeks of the intervention program, it tended (*P* = 0.096) to decline during 12 weeks of resistance exercise training, with no difference between the two groups. In addition, a tendency for a main effect of time (*P* = 0.098) was observed for time spent performing moderate intensity habitual activities. Post hoc analyses, however, did not reveal any significant differences between the different time points (Table 6).

**Table 6:** Habitual physical activity level and dietary intake assessed at baseline (pre), following 8 weeks of aerobic exercise preconditioning (AER, mid) or no exercise training (CON, mid), and after 12 weeks of subsequent resistance exercise training (post) in healthy older adults

	CON group (*n* = 17)			AER group (*n* = 17)		
	Pre	Mid	Post	Pre	Mid	Post
**Habitual physical activity**						
Step count (number per day)	8895 ± 4355	8476 ± 3192	7610 ± 3624	6978 ± 3306	7968 ± 3999	6733 ± 3389
Total active EE (kCal)	539 ± 209	518 ± 164	462 ± 151	411 ± 161	437 ± 190	379 ± 161
Sedentary time (min)	1102 ± 99	1107 ± 109	1113 ± 102	1166 ± 83	1168 ± 79	1173 ± 87
Light activity time (min)	205 ± 58	201 ± 60	211 ± 51	172 ± 50	165 ± 42	174 ± 52
Moderate activity time (min)	126 ± 54	126 ± 52	111 ± 60	104 ± 50	101 ± 48	88 ± 41
**Habitual dietary intake**						
Energy (kJ)	8245 ± 2908	8576 ± 2722	9014 ± 2218	8687 ± 2088	8317 ± 1855	7734 ± 2159
Carbohydrates (g)	207 ± 64	214 ± 71	219 ± 65	221 ± 44	223 ± 58	205 ± 55
Fat (g)	84 ± 42	83 ± 39	91 ± 29	86 ± 32	75 ± 20	69 ± 44
Protein (g)	75 ± 30	80 ± 27	80 ± 22	80 ± 23	80 ± 20	78 ± 24
Protein (g per kg bodyweight)	1.03 ± 0.45	1.14 ± 0.48	1.11 ± 0.38	1.12 ± 0.32	1.14 ± 0.29	1.08 ± 0.29

Values are mean ± SD

EE, energy expenditure

## Discussion

The present study shows that 8 weeks of aerobic exercise preconditioning increases type I and type II muscle fiber capillarization and whole-body aerobic capacity in healthy older adults. Femoral artery blood flow and vascular conductance remained unchanged both at rest and in response to exercise following aerobic exercise preconditioning. In contrast, skeletal muscle microvascular blood volume and blood flow tended to be lower in response to the standardized bout of exercise following 8 weeks of exercise preconditioning, with no changes in the control group. Despite the adaptive responses to the aerobic exercise preconditioning, no differences were observed in the gains in lean body mass, thigh muscle volume, and/or type I and II muscle fiber size between the AER and CON groups following the subsequent 12 weeks of resistance exercise training.

Although resistance exercise training is an effective treatment strategy to counteract the loss of muscle mass with age, a large inter-individual variation in the muscle hypertrophy response is observed [5]. The decline in type II muscle fiber capillarization with age has been hypothesized to restrict muscle fiber hypertrophy during prolonged resistance exercise training in older adults [6, 7]. Aerobic exercise training is the most effective exercise modality to induce angiogenesis in both young and older adults [18, 24, 25]. In support, we observed improvements in various indices of type I (range: + 17–34%) and type II (range: + 19–36%) muscle fiber capillarization following the 8 weeks of aerobic exercise preconditioning. This is in line with other studies evaluating the changes in muscle fiber capillarization in response to similar aerobic exercise training regimens in older adults [18, 24, 25], and confirms the effectiveness of aerobic exercise training to induce angiogenesis in the older population. Muscle fiber capillarization is a static measurement and provides information on the anatomical perfusion potential but does not necessarily reflect perfusion dynamics at rest or during post-exercise recovery. Therefore, we included measurements of femoral artery blood flow and muscle microvascular perfusion dynamics at rest and at multiple time points following a single, standardized bout of resistance exercise using Doppler ultrasound and CEUS, respectively. At baseline, both femoral artery blood flow and vascular conductance increased in response to exercise, peaking at *t* = 5 min and returning to baseline value at *t* = 35 min following cessation of exercise. In line, we observed a ~ 6- and ~ 20-fold increase in vastus lateralis muscle microvascular blood volume and flow, respectively, at 10 min after cessation of exercise, after which both volume and flow remained elevated up to 40 min after exercise. Previously, we have shown that low type II muscle fiber capillarization is associated with lower muscle microvascular blood volume observed at *t* = 40 min after exercise cessation in older adults [32]. Prolonged elevation of post-exercise muscle perfusion is of importance to ensure removal of accumulated waste products and allow adequate delivery of nutrients and growth factors to support muscle tissue recovery and reconditioning [8, 35, 36]. This is of particular relevance in older adults, as aging is accompanied by the loss of type II muscle fiber capillaries [15–19]. Whereas the femoral artery vascular response during post-exercise recovery remained unchanged, the vastus lateralis muscle microvascular blood volume and flow response following the same standardized bout of resistance exercise tended to be less pronounced following the 8 weeks of aerobic exercise preconditioning. This lower post-exercise microvascular blood volume and flow response likely reflects the adaptive response to the exercise preconditioning, with less metabolic perturbations following the same exercise stimulus, resulting in an attenuated perfusion response. It is interesting that the aerobic preconditioning even affects the metabolic response to a bout of resistance-type exercise. More research is warranted to further elucidate the mechanisms responsible for the interplay between structural (increase in fiber capillarization) and functional (decline in post-exercise microvascular volume and flow response) changes of the microvascular network in muscle tissue following aerobic exercise preconditioning in older adults.

Following the initial 8 weeks of aerobic exercise preconditioning, or no exercise control condition, all participants performed 12 weeks of resistance exercise training. In contrast to our hypothesis, we observed no differences in muscle hypertrophy on a whole-body, lower limb, thigh, and/or muscle fiber level following the 12 weeks of resistance exercise training between the AER and CON groups. Previously, we [7] as well as others [6] showed less muscle fiber hypertrophy during resistance exercise training in older individuals who had low muscle fiber capillarization at baseline. In addition, high muscle fiber capillarization is associated with greater acute post-exercise myogenic potential in both young and older adults [37, 38]. Hence, we hypothesized that increasing muscle fiber capillarization would augment muscle fiber hypertrophy during resistance exercise training. In support, Thomas et al. [39] applied unilateral aerobic exercise preconditioning to show greater muscle fiber hypertrophy during resistance exercise training in the leg that had been preconditioned. The greater increase in muscle fiber size in the preconditioned leg was also observed to be accompanied by an increase in muscle fiber capillarization [39]. Despite the observed improvements in the capillary network following aerobic exercise preconditioning, we failed to detect differences in muscle fiber hypertrophy between the AER and CON groups over the entire intervention period (Fig. 3). In addition, no significant correlation was observed between the aerobic exercise training-induced changes in muscle fiber capillarization and change in muscle fiber size during subsequent resistance exercise training within the AER group (data not shown). Notably, the AER group already showed a ~ 24% increase in type II muscle fiber size following the initial 8 weeks of aerobic exercise preconditioning, which is not uncommon to observe in older adults in response to aerobic exercise training [40, 41]. This initial increase in type II muscle fiber size in the AER group was, however, not statistically different from an apparent increase in the CON group, suggesting an increase in muscle fiber size during the no exercise control condition. This observation is likely a consequence of the statistical approach (i.e., two-way repeated measures ANOVA including two groups and three time points) used to evaluate the data, rather than representing actual fiber hypertrophy in the CON group. When evaluating the CON group separately, type II (as well as type I) muscle fiber size remained unchanged (*P* = 0.359) following the first 8 weeks of the no exercise control period, followed by a significant 25 ± 39% increase after 12 weeks of resistance exercise training, compared with baseline. The initial increase in muscle fiber size following the aerobic exercise preconditioning may have limited muscle fiber growth during the subsequent 12 weeks of resistance exercise training in the AER group. Furthermore, participants allocated to the CON group showed a similar increase in muscle fiber capillarization during 12 weeks of resistance exercise training compared with the 8 weeks of aerobic exercise preconditioning in the AER group. Any limitation in the growth response due to low(er) capillarization may have been compensated for by an increased capillarization as induced by the resistance training program itself. These findings suggest that the overall increase in strenuous physical activity by engaging in a structured exercise training program may be of greater importance than the specific exercise modality that is applied to induce type II muscle fiber hypertrophy and increase capillarization in an older population. This is further supported by the similar increase in aerobic capacity (VO_2peak_) over the entire 20-week intervention program in both the CON (+ 5 ± 6%) and AER (+ 6 ± 6%) groups. It should be noted that the present study was performed in healthy older males and females who were not physically impaired based on SPPB (11.5 ± 0.6 points out of 12 points), consumed sufficient dietary protein (1.0–1.1 g·kg^−1^ bodyweight), were relatively physically active (± 8000 steps per day), and were not sarcopenic based on DXA and handgrip strength (only one participant met the criteria for sarcopenia) [42]. However, most participants (27 out of 32) exhibited smaller type II compared with type I muscle fiber size, which is a clear sign of age-related muscle loss [43]. Whether similar results would be observed in more clinically compromised (sarcopenic) older adults, with cardio(micro)vascular co-morbidities, remains to be established.

As part of the larger project, we originally intended to perform additional post hoc analyses to assess whether the potential augmentative effect of aerobic exercise preconditioning on muscle fiber growth during subsequent resistance exercise training in older adults would be different in those with low(er) or high(er) baseline muscle fiber capillarization. However, the required *n* = 72 participants (*n* = 36 per group; see NTR7681) to assess this secondary research question was not feasible owing to several periods of coronavirus disease-2019 (COVID-19)-induced lockdowns and subsequent limitations during which data collection was performed. Hence, whether aerobic exercise preconditioning may have a greater impact on the muscle fiber growth response during subsequent resistance exercise training in individuals who have a particularly low muscle fiber capillarization at baseline remains to be established.

## Conclusions

Aerobic exercise preconditioning increases type I and type II muscle fiber capillarization in healthy older adults. Aerobic exercise preconditioning does not further augment muscle hypertrophy during subsequent resistance exercise training in healthy older adults. Both structural and functional microvascular characteristics following aerobic exercise preconditioning do not seem to restrict the skeletal muscle adaptive response to prolonged resistance-type exercise training in a healthy, older population.

## Supplementary Information

Below is the link to the electronic supplementary material.

**Supplemental Fig. 1:** Schematic overview of the study design. (JPG 451 KB)

## Declarations

### Data Availability Statement

The data that support the findings of this study are available from the corresponding author upon reasonable request.
