# Correction to: Aerobic Exercise Preconditioning Does Not Augment Muscle Hypertrophy During Subsequent Resistance Exercise Training in Healthy Older Adults

**DOI:** 10.1007/s40279-025-02302-6

**Published:** 2025-09-03

**Authors:** Milan W. Betz, Alejandra P. Monsegue, Lisanne H. P. Houben, Floris K. Hendriks, Janneau van Kranenburg, Thorben Aussieker, Bouke P. Adriaans, Alfons J. H. M. Houben, Lex B. Verdijk, Luc J. C. van Loon, Tim Snijders

**Affiliations:** 1 Department of Human Biology, NUTRIM Institute of Nutrition and Translational Research in Metabolism, Maastricht University Medical Centre+, P.O. Box 616, 6200 MD Maastricht, The Netherlands; 2 Cardiovascular Research Institute Maastricht (CARIM), Maastricht University, Maastricht, The Netherlands; 3 Department of Internal Medicine, Cardiovascular Research Institute Maastricht (CARIM), Maastricht University, Maastricht, The Netherlands

**Correction to: Sports Medicine** 10.1007/s40279-025-02229-y

In Fig. 1 of this article, the tick marks on the y-axes of Fig. 1F and Fig. 1L were incorrect. These have been corrected in the new Fig. 1 shown below.

**Fig. 1:**
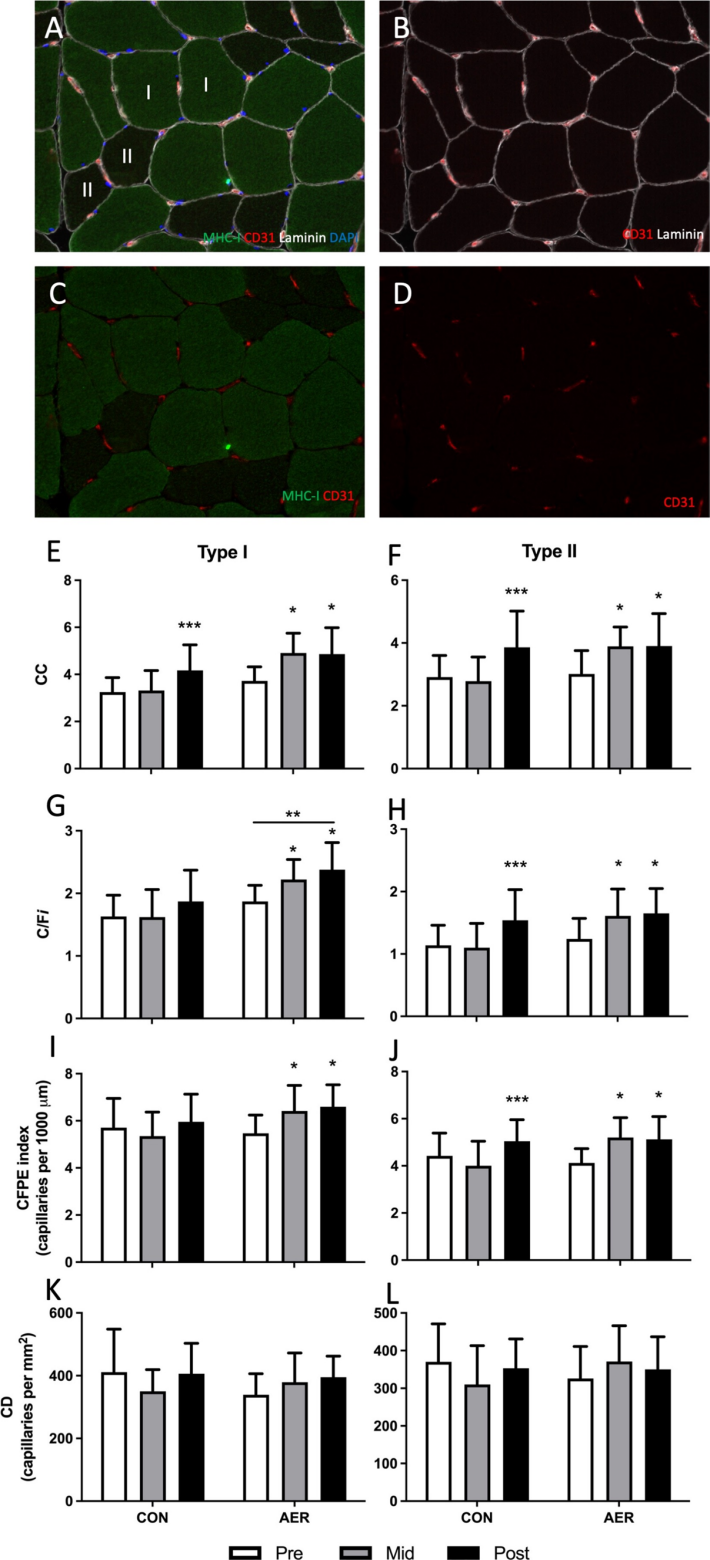
Representative images of the analyses for type I and type II muscle fiber characteristics in older adults; **A** laminin (white; cell borders), MHC1 (green; type I muscle fibers), CD31 (red; capillaries), DAPI (blue; nuclei); **B** laminin (white), CD31 (red); **C** MHC1 (green), CD31 (red); **D** CD31 (red) only. Green fibers indicate type I muscle fibers. Type I and type II capillary contacts (CC, **E–F**), capillary-to-fiber ratio (C/F*i*, **G–H**), capillary-to-fiber perimeter exchange (CFPE) index (**I–J**), and capillary density (CD, **K–L**) at baseline (pre), following 8 weeks of aerobic exercise preconditioning (AER, mid) or no exercise training (CON, mid), and following 12 weeks of subsequent resistance exercise training (post) in healthy older adults. *Significantly different compared with pre, *P* < 0.05; **significantly different compared with CON group, *P* < 0.05; ***significantly different compared with pre and mid, *P* < 0.05; data are expressed as mean ± SD

