# Supplementary figures and images for: Aerobic Exercise Preconditioning Does Not Augment Muscle Hypertrophy During Subsequent Resistance Exercise Training in Healthy Older Adults
Source: Sports Med. 2025 Apr 23;55(9):2323–38. doi: 10.1007/s40279-025-02229-y (PMC12476412; doi:10.1007/s40279-025-02229-y)

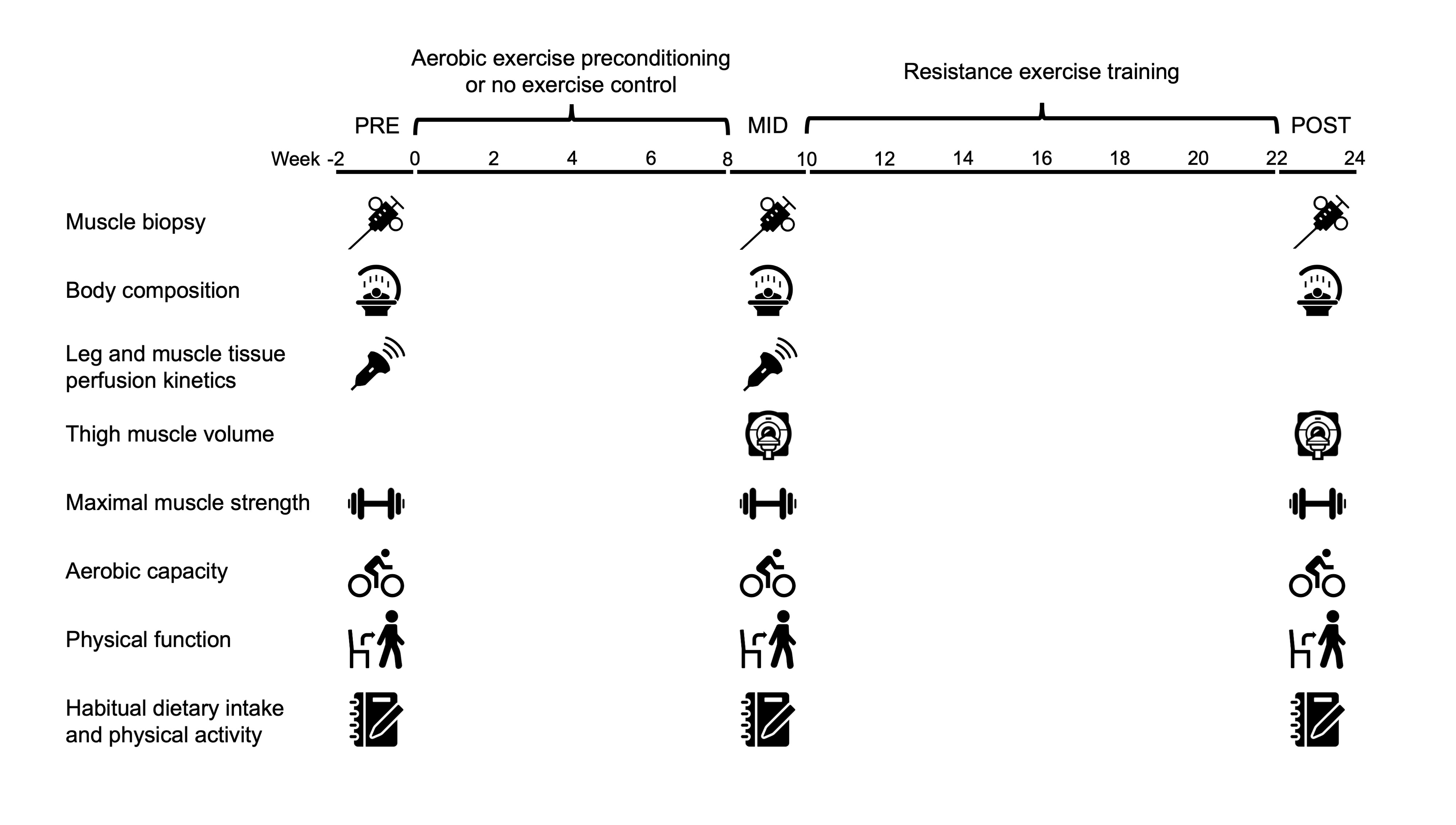

Supplement: Supplementary file 1 — Supplemental Fig. 1: Schematic overview of the study design. (JPG 451 KB) [file 40279_2025_2229_MOESM1_ESM.jpg]
